# Naming of Grammatical Classes in Frontotemporal Dementias: Linguistic and Non Linguistic Factors Contribute to Noun-Verb Dissociation

**DOI:** 10.1155/2007/428191

**Published:** 2008-03-05

**Authors:** Maria Caterina Silveri, Nicoletta Ciccarelli

**Affiliations:** Memory ClinicCentre for the Medecine of AgingCatholic UniversityRomeItaly

**Keywords:** Frontotemporal dementia, noun, verb, primary progressive aphasia, semantic dementia, dysexecutive syndrome

## Abstract

We studied noun and verb naming in three main variants of frontotemporal dementia: the frontal variant(Fv-FTD), primary progressive aphasia (PPA) and semantic dementia (SD). We further distinguished PPA in nonfluent and fluent forms and restricted diagnosis of SD to subjects with progressive semantic breakdown leading to agnosia for words and objects. Fv-FTD and nonfluent-PPA named objects better than actions, SD showed an inverse dissociation and no specific pattern emerged in fluent-PPA. In this last group, in spite of the broad definition of fluent aphasia, quite heterogeneous patterns of language disorders and word class dissociation emerged when single-subject analyses were performed. In fv-FTD correlations between executive tasks and action naming were stronger than between executive tasks and object naming. We conclude that both linguistic and non linguistic factors, in particular an executive deficit, contribute to grammatical class dissociation. We also suggest that the fluent vs. nonfluent distinction does not reflect the complexity of primary aphasia.

## 1. Introduction

Selective impairment of grammatical classes of words has been consistently demonstrated in patients with brain lesions and specific anatomical substrates have been hypothesised for elaboration of nouns and verbs. Production of nouns is limited in patients with posterior lesions, while anterior lesions generally impair production of verbs [8,13,18,29,35,56]. This observation mostly drawn from stroke patients led to the hypothesis that the neural substrates critical for noun and verb processing are located in the temporal and frontal regions respectively, with a few exceptions suggesting the extension of the neural network involved in verb processing to the parietal lobe [1,49,50].

Evidence of grammatical class dissociation extends to patients affected by degenerative brain pathology. Also in this case, observations converge in suggesting involvement of the anterior vs. posterior regions in subjects with selective deficit in naming either verbs or nouns [5,6,14,23,26,30,38,51,52]. Nevertheless, dissociation in patients with degenerative brain disease present some peculiarities. For example, the general observation that verbs are more "difficult" than nouns acquires special evidence. Group studies on dementia patients indicate, in fact, that in two of the most frequent forms of degenerative dementia, Alzheimer's disease (AD) and the frontal variant of frontotemporal dementia (FTD), naming verbs is more impaired compared to naming nouns [7,27,52]. Similarly, Rhee et al. [39] found a disproportionate deficit for verbs in patients presenting with various subtypes of frontotemporal dementia. The more severe impairment for verbs was confirmed not only in the frontal variant but also in

ISSN 0953-4180/07/$17.00 © 2007 – IOS Press and the authors. All rights reserved patients with primary language disorders, both fluent and nonfluent. A more recent study on patients affected by primary progressive aphasia (PPA) seems at variance with this view [22]. In fact, only patients with evidence of anterior damage (nonfluent aphasics and FTD patients with motoneuron disease), showed a selective impairment in naming verbs. Fluent aphasics were, on the contrary, more impaired in the word-class nouns. Therefore, verbs are generally penalised compared to nouns in dementia, but there are exceptions.

According to Gentner [17], verbs express relational concepts compared to nouns that are defined in terms of object-related concepts. Thus, since verbs are implemented by elaboration of a wider range of information compared to nouns, a greater amount of executive resources is presumably requested. If this is true, the impairment of verbs should become evident in pathological conditions that reduce executive resources and particularly in dementia. On the contrary, nouns would seem selectively damaged mostly when the degenerative process selectively involves the posterior perisylvian areas of the dominant hemisphere and an aphasic disorder is clinically evident.

In other words, what we observe in patients with degenerative brain diseases could be various combinations of two effects: higher sensitivity to neural damage of one grammatical class (verbs) and selective involvement of cortical areas critically involved in processing specific classes of words [52].

We studied populations of subjects affected by three variants of frontal dementia supposed to differentially affect brain regions crucial for executive abilities and language: the frontal variant (fv-FTD), primary progressive aphasia (PPA) and semantic dementia (SD). Within the subgroup of patients with PPA, we tried to distinguish nonfluent from fluent forms in which the patterns of grammatical class dissociation is expected to be different. The distinction between nonfluent and fluent forms, however, may not be trivial in primary aphasias. Nevertheless, a rough distinction can be attempted on the basis of standard clinical criteria such as fluency, articulation, grammatical construction and comprehension [32].

Subclassification of primary aphasias may be complex not only due to their intrinsic variability, but also due to some inconsistency in the current terminology. For example, the term semantic dementia is sometimes used to designate fluent aphasia with comprehension disorders, while it should be restricted to the syndrome typically dominated by a progressive and selective semantic degradation that in later stages leads to agnosia for objects and words [24,36,53,54]. Fluent forms that do not fit descriptions for semantic dementia can be however, observed. Sometimes in fact, language disorders also involve the phonological level, and the comprehension deficit may not be generated exclusively by the semantic decay [10,32].

We selected patients in whom linguistic disorders of at least two years duration were the salient feature [31,32]. We classified as nonfluent PPA patients with ipofluent speech, agrammatism, reduced phrase length and rate of utterance, good comprehension of conversational speech and ipoarticulation. Patients with fluent speech, normal articulation, paraphasias (phonemic or semantic) and poor comprehension were considered as fluent PPA. We restricted the diagnosis of SD to patients who presented a progressive and selective breakdown of semantic knowledge that made them unable to understand the meanings of words and objects, without any evidence of a true aphasic disorder. We also studied a population of fv-FTD patients.

We expected a disproportionate verb-naming deficit in subjects with nonfluent PPA in which a distribution of the damage in anterior brain regions could be presumed, and in subjects with fv-FTD that are supposed to be affected by dysexecutive disorders but not by aphasia. Conversely, we expected a more severe noun-naming impairment in fluent PPA and in SD. However, since these two subgroups of patients, although both affected by "fluent" language disorders, differed for clinical expression (and presumably for distribution of neural damage within the posterior brain regions) [9,43], we did not expect their word-class dissociation pattern to be necessarily homogeneous. In order to look for possible dissociation between phonological and orthographic lexicons of nouns and verbs [22], both the oral and written modality were explored in patients with PPA and SD.

If our expectations were confirmed, the hypothesis that noun and verbs are elaborated by different neural substrates would receive further support by evidence drawn from degenerative brain pathology. At the same time we could also corroborate the hypothesis that noun verb dissociation is multiply determined and that an executive deficit concurs in verb naming deficit.

## 2. Method

### 2.1. Subjects

We examined 59 subjects who met the general clinical criteria for different subgroups of frontotemporal

Table 1
Neuropsychological examination in the four subgroups of FTD (mean and standard deviations of the adjusted scores and the cut-off are reported in each task)

| | Fv-FTD | NonFluent-PPA | Fluent-PPA | SD | Cut-off |
|---|---|---|---|---|---|
| Immediate free recall | 28.78 (8.25) | 22.36 (11.47) | 15.32 (8.70) | 22.51 (11) | 28.53 |
| Delayed free recall | 4.96 (3.04) | 5.49 (3.50) | 3.48 (2.86) | 3.27 (2.11) | 4.69 |
| Forced recognition | 0.83 (0.15) | 0.90 (0.09) | 0.84 (0.12) | 0.84 (0.06) | 0.90 |
| Verbal span forward | 4.90 (0.80) | 4.14 (1.25) | 3.88 (0.64) | 6 (1.55) | 7+−2 |
| Verbal span backward | 2.50 (1.33) | 2.66 (1.30) | 2.29 (1.80) | 3.67 (0.82) | 5+−2 |
| Spazial span forward | 2.92 (1.93) | 3.90 (1.40) | 4.33 (0.52) | 5.75 (0.50) | 7+−2 |
| Spatial span backward | 2.32 (1.44) | 2.87 (1.06) | 2.83 (0.41) | 4.50 (0.58) | 5+−2 |
| Letter fluency | 17.90 (11.42) | 17.14 (9.54) | 6.96 (10.96) | 19.10 (3.15) | 17.35 |
| Semantic fluency | 8.12 (4.08) | 8.04 (3.99) | 6.17 (3.16) | 5.06 (2.63) | 16.76+−4.38 |
| Double barrage (accuracy) | 0.88 (0.11) | 0.97 (0.03) | 0.82 (0.36) | 0.97 (0.03) | 0.92 |
| Rey-Osterrieth-figure copy | 17.89 (11.11) | 24.61 (10.23) | 17.5 (13.09) | 33.61 (2.40) | 28 |
| Oral praxis | 9.20 (1.03) | 8.75 (1.16) | 9.06 (1.87) | 9.50 (0.84) | 9 |
| Limb praxis | 9.40 (0.72) | 8.79 (1.97) | 9.38 (1.06) | 8.83 (1.17) | 9 |
| Raven | 18.95 (6.84) | 23.71 (7.14) | 18.59 (7.12) | 27.99 (3.90) | 19.96 |
| Trial making test (error) | 11.27 (10.77) | 2.88 (3) | 15 (15.62) | 2 (0) | 1.09+−1.30 |
| Trial making test (time) | 459.82 (303.35) | 220.25 (71.61) | 650.67 (226.32) | 155.25 (73.10) | 180.09”+−68.80” |
| WCST (perseverations) | 19.85 (12.53) | 11.62 (9.06) | 22.25 (5.50) | 9.60 (12.10) | 10.59+3.69 |
| Stroop (time) | 100.09” (139.08) | 123” (70.35) | 209.80” (262.30) | 66.25” (52.62) | 74.27”+−50.70” |
| VOSP (incomplete letters) | | | | 15.50 (4.20) | 16 |
| TEA (telephone search) (time) | 12.60” (10.75) | 11.55” (6.17) | 9.98” (3.22) | 9.55” (2.05) | 4.22”+−1.5” |
| TEA (telephone search while counting) (time) | 41.27” (77.24) | 20.58” (29.35) | 46.61” (66.09) | 18.75” (1.89) | 2.28”+−2.8” |
| BORB (item match) | | | | 30.60 (1.52) | 30+−2.2 |
| BORB (association match) | | | | 21.20 (4.87) | 27.5+−2.4 |
| BORB (foreshortened match) | | | | 22 (1.22) | 21.6+−2.6 |
| BORB (minimal feature) | | | | 23.141 | 23.3+−2 |
| Naming | | 65 (12.81) | 43 (18.57) | 26.50 (13.03) | 61 |
| Lexical comprehension | | | | 125.33 (18.23) | 147 |
| Benton facial recognition test | | | | 37.50 (7.87) | 38 |
| Face familiarity check (errors) | | | | 2.77 (1.63) | 2.05 |
| Face identification (errors) | | | | 5.18 (2.68) | 2.99 |
| Repetition | | 43 (2.97) | 41.75 (3.84) | 44.20 (1.79) | 43 |
| Reading | | 88.40 (4.81) | 83.25 (6.27) | 88.60 (6.07) | 90 |
| Sentence comprehension | | 51 (10.30) | 47.25 (4.73) | 55.20 (7.12) | 58 |
| Lexical comprehension | | 38.92 (1.32) | 35.50 (2.56) | 33.14 (3.67) | 38 |
| Comprehension of conversational speech | | +++ | +−− | +++ | |
| Speech fluency | | +−− | +++ | +++ | |
| Articulation | | +−− | +++ | +++ | |
| Phrase length | | +−− | ++− | +++ | |
| Paraphasias | | +−− | +++ | +−− | |

dementia [37]: 30 fv-FTD (15 men and 15 women); 22 PPA (14 men and 8 women) and 7 semantic dementia (5 men and 2 women). All subjects underwent complete laboratory investigations, physical and anamnestic examinations, MRI and/or CT-scan. Most patients underwent a functional study (SPECT).

The general neuropsychological examination included memory tasks (Rey's immediate and delayed free-recall of 15 words, Rey Osterrieth figure delayed recall), visuospatial tasks (line cancellations), letter and semantic fluency, Raven's Coloured matrices, digit and spatial span and tasks of oral, constructional and limb praxis. All subjects also underwent a formal examination of the executive ability (Stroop task, WCST, Trial Making test), attention (subsets of the Test of Everyday Attention-TEA) [41] and behaviour (NeuroPsychiatry Inventory, NPI) [12]. Language was explored by the tests of the BADA [34]. Visual recognition deficit in SD was explored by means of The Visual Objects and Space Perception Tasks (VOSP) [25] and by subsets of the Birmingham Objects Recognition Battery (BORB) [40]. Face processing was examined by the Benton Facial recognition Task (BFRT) [4] and by the Familiarity check and Face identification tasks [16]. Results are summarized in Table 1. In subjects with PPA, samples of speech were recorded for further evaluation and aphasia classification, according to the criteria above described. Some patients with fv-FTD presented with verbal inertia, but none had aphasic disorders or evidence of agrammatic production.

Table 2
Main demographic characteristics and general severity of mental deterioration (MMSE) and naming deficit (mean. standard deviation and range)

| | Age (years) | Education (years) | Duration (months) | MMSE | Oral naming (correct/56) |
|---|---|---|---|---|---|
| Fv-FTD | 73.87 (7.27) | 8.87 (5.04) | 30.40 (19.10) | 23.70 (3.52) | 43.30 (8.94) |
| ($n = 30$) | 55–91 | 5–17 | 12–96 | 18–30 | 19–54 |
| Nonfluent | 70.17 (9.94) | 11.86 (4.70) | 35 (15.23) | 24.79 (3.81) | 40.36 (12.95) |
| PPA ($n = 14$) | 51–87 | 5–19 | 24–72 | 18–29 | 10–54 |
| Fluent PPA | 73.58 (5.43) | 12.66 (3.42) | 36 (12.3) | 17.75 (7.07) | 17.25 (13.96) |
| ($n = 8$) | 65–83 | 8–17 | 24–60 | 9–25 | 7–47 |
| SD | 68.26 (9.32) | 13.42 (5.028) | 42.85 (16.76) | 24.29 (4.19) | 28.15 (11.27) |
| ($n = 7$) | 50–77 | 5–17 | 24–72 | 17–29 | 14–44 |

### 2.2. Experiment

Naming of objects and actions was examined by means of the BADA oral and written naming tasks [34]. In these tasks, nouns and verbs denoting objects and actions are matched for word frequency, length and phonological complexity. The oral naming tasks consist of 28 objects and 28 actions, the written naming tasks of 22 objects and 22 actions. In both tasks the pictures, depicted in black and white, are presented one a time by the examiner. The patient is requested to name or to write the name or the verb denoting the picture. The pictures are presented in fixed order in each task. The four tasks (oral naming of objects and actions and written naming of objects and actions) are presented in random order. Fv-FTD patients performed only the oral naming tasks.

### 2.3. Statistics

The Wilcoxon matched pairs test (Z) was used to compare dependent samples (variables) (different word classes or modalities within groups). The Kruskal-Wallis test and the multiple comparisons of mean ranks were adopted for multiple comparisons across independent samples (groups). Pairs of independent samples were compared using the Mann-Whitney U-test. Frequency distribution was tested using the Chi-square statistic and the Fisher exact test. Correlations were explored by means of the Spearman Rank R.

### 2.4. Group characteristics

In PPA and SD patients at least one of the brain imaging studies documented an asymmetrical cerebral atrophy, for involvement of the left perisylvian areas. In all subjects who met the criteria for fv-FTD, the brain imaging showed the involvement, although sometimes non selective, of the frontal lobe and no damage in the deep temporal regions. The physical examination was negative in all fluent PPA and SD patients. One SD patient developed a Kluver-Bucy syndrome. In the nonfluent forms, one patient presented moderate cogwheeling on the right side of the body. No other patient in the various subgroups developed neurological signs during the time course of the examination.

The main demographic data, disease duration and MMSE of the various subgroups are reported in Table 2. Groups of patients did not differ for age. SD had higher education than fv-FTD (Mann-Whitney U test = 2.074; $p = 0.0038$). Fluent PPA had significantly lower scores on the MMSE compared to nonfluent PPA (Mann-Whitney U test = 22.50; $p < 0.022$) and tended to obtain lower scores than SD (Mann-Whitney U test = 11.50; $p = 0.056$). Patients with PPA and SD preserved a high degree of autonomy in everyday living activities.

Since the MMSE is not a reliable measure of general cognitive deterioration in subjects with verbal disorders (and also in fv-FTD), we assumed that clinical comparisons across groups would be better expressed by disease duration (number of months from onset at the time of the examination) and by general severity of naming disorders independently of grammatical class effects. Groups did not differ for disease duration. Multiple comparisons of oral naming across groups were significant (Kruskal-Wallis test: $H_{(3,N=59)} = 19.6724$; $p = 0.0002$; fv-FTD > than fluent PPA ($p = 0.0007$) and SD ($p = 0.0380$); nonfluent PPA > than fluent PPA ($p = 0.0120$). Similarly, multiple comparisons across groups were significant in written naming (Kruskall-Wallis test: $H_{(2,N=25)} = 9.3011$; $p = 0.0096$; nonfluent PPA > than fluent PPA ($p = 0.0078$); nonfluent PPA = SD; fluent PPA = SD).

## 3. Results

The results obtained by the groups of patients in oral and written naming of nouns and verbs are summarised

Table 3
Performance of patients subgroups in naming nouns and verbs in oral and in written modality (mean, standard deviation. and range)

| | Oral naming | | Written naming | |
|---|---|---|---|---|
| | Noun ($N = 28$) | Verb ($N = 28$) | Noun ($N = 22$) | Verb ($N = 22$) |
| Fv-FTD | 24.37 (3.61) | 18.87 (5.66) | – | – |
| | 13–28 | 6–27 | | |
| Nonfluent PPA | 22.36 (6.71) | 18.00 (6.58) | 17.00 (6.58)* | 15.50 (5.95)* |
| | 6–28 | 4–26 | 5–22 | 4–22 |
| Fluent PPA | 8.25 (8.96) | 9.00 (5.13) | 6.43 (6.48)** | 7.43 (5.44)** |
| | 1–21 | 5–20 | 0–20 | 2–15 |
| SD | 10.86 (6.39) | 17.29 (5.26) | 8.67 (4.03)*** | 14.00 (4.57)*** |
| | 4–21 | 10–23 | 4–15 | 7–21 |

*12/14 subjects.
**7/8 subjects.
***6/7 subjects.

in Table 3.

### 3.1. *Between-group analysis (fv-FTD, nonfluent PPA, fluent PPA, SD)*

In spite of approximately the same disease duration, fluent PPA had a more severe cognitive impairment (MMSE) than other groups and lower performance in naming. Thus, across-group differences were to a large extent generated by the different severity of the naming impairment and not by selective decay in one grammatical class. Anomia, in fact, was more severe in fluent than in nonfluent aphasics. As expected, nonfluent and fv-FTD patients named nouns better than fluent patients (noun oral naming: Kruskall-Wallis test (H $_{(3,N=59)}$ = 23.8609 $p = 0.0000$; fv-FTD > than SD (0.0031) and fluent PPA (0.0006); nonfluent PPA > than fluent PPA (0.0122) and SD (0.0321). However, verbs were also more impaired in fluent PPA and SD than in fv-FTD and nonfluent PPA (oral naming of verbs: Kruskall-Wallis test ($H_{(3,N=59)} = 11.9286$; $p = 0.0076$; nonfluent PPA > than fluent PPA ($p = 0.0400$); fv-FTD > than fluent PPA ($p = 0.0037$).

A similar pattern of results could be observed in the written modality: nonfluent PPA named nouns better than fluent PPA and no significance emerged between nonfluent PPA and SD and between fluent PPA and SD (nouns: Kruskall-Wallis test (H $_{(2,N=25)}$ = 11.5633; $p = 0.0031$; nonfluent PPA > than fluent PPA (0.0046); nonfluent PPA = SD ($p = 0.0749$); SD = fluent PPA). However, naming verbs was also significantly better in nonfluent PPA than in fluent PPA (Kruskall-Wallis test (H $_{(2,N=25)}$ = 7.6007; $p = 0.0224$; nonfluent PPA > fluent PPA ($p = 0.0186$); nonfluent PPA = SD; fluent PPA = SD).

### 3.2. *Within-group analysis*

1) *Noun verb dissociation in oral naming* (*all groups*)

In patients with "anterior" damage, the expected dissociation, i.e., with nouns significantly more preserved than verbs, was confirmed (Fv-FTD: Z = 4.60; $p <$ 0.0000; nonfluent PPA: Z = 3.18; $p < 0.0152$). Conversely, SD patients proved to be more impaired on nouns than verbs (Z = 2.37; $p < 0.018$). No difference emerged in fluent PPA between nouns and verbs.

2) *Noun verb dissociation in written naming* (*nonfluent PPA, fluent PPA, SD*)

Also in the written modality, nonfluent PPA named nouns significantly better than verbs (Z = 2.43; $p <$ 0.015) and SD patients named verbs significantly better than nouns (Z = 2.20; $p < 0.028$). The difference did not reach significance in fluent PPA.

### 3.3. *Cross-modality comparisons*

Oral naming was better than written naming in all groups for both nouns and verbs, but no difference reached significance.

### 3.4. *Single-subject analysis*

Twenty-eight out of 30 fv-FTD obtained lower scores in naming verbs than nouns (two obtained the same score). All but one nonfluent PPA (13/14) obtained lower scores in naming verbs than nouns in oral naming (one obtained the same score); in written naming, eight out of 12 patients (two patients did not perform the task) named nouns better than verbs; two obtained the same score and only one patient scored one point higher

Table 4
Main features of the aphasic disorder in eight patients with different subtypes of fluent PPA

| Type of aphasia | TCSA | | | Ph-Patients | | WA | | |
|---|---|---|---|---|---|---|---|---|
| | 1 | 2 | 3 | 4 | 5 | 6 | 7 | 8 |
| Semantic errors in production* | +++ | +++ | ++ | ++ | + | ++ | + | +++ |
| Phonological errors in production* | − | − | | ++ | +++ | − | − | − |
| Lexical comprehension (word-picture matching) (correct responses) | 35/40 | 31/40 | 33/40 | 38/40 | 38/40 | 36/40 | 38/40 | 35/40 |
| Syntactic comprehension (sentence-picture matching) (correct responses) | 49/60 | 37/60 | 48/60 | 49/60 | 47/60 | 45/60 | 53/60 | 50/60 |
| Word reading (correct responses) | 87/92 | 79/92 | 84/92 | 71/92 | 80/92 | 90/92 | 88/92 | 87/92 |
| Stress errors in reading** | 2 | 3 | 4 | 0 | 0 | 0 | 0 | 0 |
| Word repetition (correct responses) | 45/45 | 44/45 | 45/45 | 34/45 | 38/45 | 43/45 | 43/45 | 42/45 |

TCSA = Transcortical sensory aphasia.
Ph-Patients = patients with phonological disorders.
WA = Wernicke aphasia.
*Production = spontaneous speech and naming.
**In Italian language stress errors indicate "surface" reading.

on verbs than on nouns. All SD patients named verbs better than nouns in both the oral and written modality.

Fluent PPA did not show significant dissociation between nouns and verbs in either modality. A rather heterogeneous pattern consistently emerged in this subgroup of patients. Only three patients out of 8 named verbs better than nouns in both oral and written modalities (in one patient the written modality could not be explored).

In order to investigate the nature of these unexpected results in fluent PPA, we performed a detailed analysis of each patient's linguistic deficit. In spite of their classification as fluent aphasics, the linguistic patterns were quite different in the various subjects (Table 4). Three had a disorder that could be classified as transcortical sensory aphasia (TCSA) with paraphasic production, good repetition and very poor comprehension of both spoken and written language; in two patients the salient feature was the presence of phonological errors (Ph-patients) in production tasks, including repetition, but low comprehension did not allow classifying them as proper conduction aphasia patients (logopenic aphasia of other classifications? [19]); three patients had a semantic disorder that was similar to but not as selective as the one reported in semantic dementia and rather similar to the classical description of Wernicke's aphasia (WA). In fact, they presented not only anomia, paraphasias and comprehension disorders, but also errors in transcoding tasks. The frequency of the correct responses in the two grammatical classes was reconsidered in these three subgroups. All patients with TCSA named verbs better than nouns in the oral modality. Only two patients performed the written naming test, and both of them named verbs better than nouns. The total distribution of the correct responses was significant in the oral modality (verbs vs. nouns = 18/84 vs. 6/84; Chi-square$_{(df=1)}$ = 7.00; $p = 0.0082$) and a tendency was also confirmed in the written modality (verbs vs. nouns = 10/44 vs. 3/44; Yates corrected-Chi-square$_{(df=1)}$ = 3.25; $p = 0.0715$). Both Ph patients showed the opposite pattern in both modalities (oral naming: verbs vs. nouns = 29/56 vs. 40/56; Chi-square$_{(df=1)}$ = 4.57; $p = 0.0326$; written naming: 22/44 vs. 26/44; Yates corrected-Chi-square$_{(df=1)}$: 0.92, ns; oral + written: verbs vs. nouns = 51/100 vs. 66/100; Chi-square$_{(df=1)}$ = 4.63; $p = 0.0314$). Distribution was far from significant in WA patients (oral naming: verbs vs. nouns = 25/84 vs. 20/84; written naming: verbs vs. nouns = 20/66 vs. 16/66; oral + written: 45/150 vs. 36/150). In this subgroup, 2/3 patients scored better on verbs than on nouns in the oral modality and 2/3 scored better on nouns than on verbs in the written modality.

No patient with PPA or SD presented a dissociation between oral and written modalities.

### 3.5. *Relationship between executive abilities and verb production in fv-FTD*

Table 5 shows correlations between different measures of executive ability (Phonological Word Fluency, Rey-Osterrieth Figure copy, Trial Making test-errors, Trial Making test-time, Stroop-errors, Stroop-time, WCST-perseverations) and noun and verb naming. Although executive tasks tended to be significantly correlated with both grammatical classes, correlations with verb naming were stronger than with noun naming.

Table 5
Correlation (Spearman Rank R) between tasks of executive ability and naming of nouns and verbs

| | Naming noun | | Naming verb | |
|---|---|---|---|---|
| Phonological word fluency | 0.4123 | * | 0.5442 | * |
| Rey figure copying | 0.5048 | * | 0.6180 | * ° |
| Trial making test-errors | −0.5248 | * | −0.6343 | * ° |
| Trial making test-time | −0.4982 | * | −0.6196 | * ° |
| Stroop-errors | −0.2683 | | −0.3741 | * |
| Stroop-time | −0.1366 | | −0.0629 | |
| WCST (perseverations) | −0.1178 | | −0.1249 | |

$^{*}p = 0.05$; $^{\circ}p = 0.001$.

## 4. Discussion

Our expectations were confirmed. Noun deficit was clear in subjects with SD and verb deficit as well was clear in fv-FTD and non fluent aphasics. Individual analysis confirmed that the pattern of dissociation was consistent across subjects with fv-FTD, nonfluent PPA and SD. Patients with fluent PPA, on the contrary, did not show the expected impairment in naming nouns compared to verbs; in fact, they did not show any specific pattern of dissociation as a group. However, when a detailed analysis of the aphasic disorder was made, patients of this group had quite heterogeneous linguistic deficits in spite of the broad definition of fluent aphasia: three had transcortical sensory aphasia, two a phonological disorder and three had Wernicke-like aphasia. While transcortical sensory aphasics showed a disproportionate deficit for nouns, patients with phonological disorders revealed the opposite pattern, with a disproportionate deficit in naming verbs. No specific pattern emerged in subjects with Wernicke's aphasia.

There was no evidence of a cross-modality dissociation between nouns and verbs in our patients; written naming was consistently more impaired than oral naming in all groups of PPA and SD patients independently of the grammatical class. However, the phonological/orthographic regularity of the Italian language could prevent the emergence of dissociation due to the selective impairment of the phonological or orthographic lexicons, since the spelling can be obtained by using the sublexical procedures.

Different orders of considerations may be drawn from these data: 1) disorder of verb naming may be observed in anterior brain lesions, but it is not necessarily an expression of an aphasic deficit; fv-FTD are not aphasic, yet their deficit for verbs is clear. This finding confirms that the anterior regions are crucial for verb processing but also suggests that an account of the grammatical class dissociation exclusively based on the hypothesis of a linguistic deficit is not tenable since verbs are also impaired in subjects whose language is virtually intact. At the same time, confirming previous evidence in subjects with stroke [1,49,50], also in primary linguistic disorders a verb deficit may be generated by posterior, possibly parietal lesions as suggested by the presence of a phonological disorder classically attributed to parietal damage. The involvement of the supramarginal gyrus has been reported in PPA, presenting as conduction aphasia [21]. At the same time, a parietal involvement has been demonstrated in so-called logopenic progressive aphasia [2,19], in which well articulated but reduced speech is associated with low comprehension but not with semantic deficits. 2) The clinical expression of linguistic disorders in primary aphasias is large, and the nonfluent vs. fluent dichotomy is a very broad distinction that does not account for the complexity of the observations when detailed analyses of the linguistic disorder are performed.

### 4.1. *Aphasia, site of lesion and word class deficit*

If impaired verb production can be consistently demonstrated in subjects without evidence of aphasia, such as fv-FTD [7,52], or in subjects with motoneuron disease, in which the language disorder is strongly dominated by "peripheral" motor components [3,22], a linguistic interpretation of the deficit (lexical, semantic or morphological) cannot fully account for the reduced ability to produce verbs. Aphasia, as well as left-hemisphere damage with specific involvement of the language neural substrates, is not a typical feature of this pathology. In our fvFTD group, the disproportionate impairment for verbs was quite consistent across subjects in the absence of any evidence of asymmetrical (left) distribution of the damage. In addition naming verbs (but not naming nouns) significantly correlated with tasks exploring executive abilities. All of these observations support the hypothesis that the frontal lobe *per-se*, and not only the left frontal areas, could be implicated in verb production.

In this view, the frontal lobe would contribute to processing both linguistic or non-linguistic aspects of verbs. The left frontal areas have traditionally been related to the deficit for verbs by authors who postulate the lexical hypothesis [8,35] or who interpret the selective damage for verbs as an expression of a syntactic [44] or morphological disorder [45,55]. The left frontal areas have also been considered crucial for the conceptual representation of action, to the extent that these regions would implement features of motion and motor planning [13,30].

The frontal lobe, however, is also the structure that implements planning and control functions, and frontal lobe lesions are typically expected to reduce the executive resources necessary to perform complex tasks. Thus, the deficit for the class verbs in frontal patients could also be due to a reduced processing resource that becomes mostly evident in relation to the different task complexity, e.g., action naming compared to object naming. Verbs are "harder" than nouns (badly remembered, longer to acquire, less imageable, less stable in translation between languages, acquired later . . . ) since they express relational concepts, and in perceptual as well as in conceptual domains their elaboration is supposed to require manipulation of a greater amount of information [17]. Ultimately, the left frontal areas are crucial for the linguistic elaboration of verbs (lexical, semantic, syntactic and morpho-phonological), but the frontal lobe as such is also crucial for processing non linguistic components. The complexity of verb compared to noun is also suggested by the extension of the cerebral areas implicated in their processing that include not only left-frontal, and in general frontal areas, but also left parietal regions. In our sample of fluent PPA, a disproportionate verb deficit was associated with a phonological disorder and, consistently with previous reports, this suggests a left parietal involvement [1,49,50]. Also in relation to connections with the left frontal lobe, these regions are probably relevant for the morpho-phonological processes crucial for verb elaboration [45,55] as well as for the somatosensory aspects of motion [13,48] and the representation of spatial relations between objects [46].

On the contrary, the pattern of results showed by SD patients is quite consistent in suggesting a proper semantic deficit. By definition, SD is dominated by a progressive degradation of the knowledge that in later stages leads to agnosia for objects and words, due to impairment of the anterior left temporal regions. Thus, in SD patients the deficit for nouns may be interpreted as the typical expression of a semantic memory breakdown. Verbs are, on the contrary, largely spared in this pathology confirming that the processing of the verb is to some extent independent of semantic memory.

The noun impairment showed by the TCSA was not surprising since this subgroup of aphasics might represent "less pure" forms of SD. In fact, compared to SD patients TCSA had semantic paraphasic production and a more severe impairment in understanding conversational speech. At the same time, they cannot simply be considered as more severe forms of SD given the comparable disease duration (indeed, disease duration tended to be even longer in SD). Last, Wernicke aphasics probably represent an aphasic population in which deficits in multiple linguistic domains prevent the emergence of clear word class dissociation. Finally, a semantic deficit largely accounts for the selective impairment for nouns, while multiple factors likely contribute to verb impairment.

### *4.2. PPA as a symptom complex*

Our results suggest that classification of the primary aphasias in fluent vs. nonfluent forms might be an oversimplification, and that identification of finer-grained syndromes within this broad distinction should be attempted [20]. The pattern of noun verb dissociation allows discovering heterogeneity within the so-called primary fluent aphasias. In our sample of patients, fluent aphasia assumed different characteristics in the various subjects. Thus, the conclusion that patients with fluent PPA have a disproportionate disorder with word class nouns should be reconsidered: fluent aphasics have difficulty naming nouns when, in the broader definition of fluent aphasia, they fit the criteria for semantic dementia or, more generally, transcortical sensory aphasia. Robinson et al. [42] reported a similar case with a selective sparing of verbs. Although the patient was diagnosed on clinical grounds as having Alzheimer's disease (but see Davies et al. [15], on the lack of correlation between clinical evidence and pathological findings in fluent PPA), this report confirms that verbs may be selectively spared in primary linguistic disorders when they assume the characteristics of fluent aphasia. When fluent aphasics present with phonological disorder the category of verbs may be disproportionally impaired, as already demonstrated in stroke patients [49,50]. At the same time, the presence of a semantic deficit seems not sufficient for the emergence of a disproportionate noun impairment if the semantic deficit is not selective, but it is a component of a more complex aphasic syndrome, as in our population of Wernicke-type aphasics.

Nonfluent aphasics seem to present quite a consistent pattern of dissociation with selective verb impairment. It is likely that multiple phenomena, such as impairment of syntactic and morphological components of language elaborated in left frontal areas, decay of motor representations of action verbs as well as limitation of executive resources typical of frontal lobe damage, concur to generate the verb deficit in this population of aphasics.
